# Cumulative, collective or conservative? A review of seven decades of writing about women, gender, sex, sexuality and intersectionality in international and comparative education

**DOI:** 10.1007/s11159-025-10193-y

**Published:** 2025-11-04

**Authors:** Elaine Unterhalter

**Affiliations:** Department of Education, Practice and Society, Institute of Education, University College London, London, UK

**Keywords:** Gender equality and education, Women’s education, Women researchers, Men researching gender, Intersectionality, Sexuality and education, International and comparative education

## Abstract

This article traces the cumulative and collective development of knowledge on women, gender, sex, sexuality and intersectionality over seven decades of scholarship in international and comparative education. The discussion centres on a history of articles published in the *International Review of Education (IRE)*, written by women and  by women and men about women’s learning and, gender, sex, sexuality , intersectionality and education*.* This frames an analysis of how the scholarship  on these themes has been discussed, contested or silenced in the field of international and comparative education. The analysis draws out themes and considers how they link to the work of United Nations organisations and movements for social change. In an age of vulnerability, stark inequalities, powerful attacks on the concept of gender and evident planetary boundaries with regard to resources, the article highlights the significance of educational engagement with the politics of gender, the many forms these connections take and the potential for forming alliances. Building significant support for human development and equity as these values come under attack requires paying close attention to insights from data on gender, sex, sexuality and intersectionality, their conceptual framings, combined with empathetic understanding and reflective practice to help form solidarities.

**Supplementary Information:**

The online version contains supplementary material available at 10.1007/s11159-025-10193-y.

## Résumé

Cumulatif, collectif ou conservateur ? Une revue de sept décennies d’écrits sur les femmes, le genre, le sexe, la sexualité et l’intersectionnalité dans l’éducation internationale et comparative – Cet article retrace l’évolution cumulative et collective des connaissances sur les femmes, le genre, le sexe, la sexualité et l’intersectionnalité au cours de sept décennies de recherche en éducation internationale et comparative. La discussion porte sur l’historique des articles publiés dans la *Revue internationale de l’éducation (RIE)*, écrits par des femmes et des femmes et hommes et traitant de l’apprentissage des femmes, du genre, du sexe, de la sexualité, de l’intersectionnalité et de l'éducation. Cela permet d’analyser la manière dont les recherches sur ces thèmes ont été discutées, contestées ou passées sous silence dans le domaine de l’éducation internationale et comparée. L’analyse met en évidence certains thèmes et examine leur lien avec le travail des organisations des Nations Unies et les mouvements en faveur du changement social. À une époque marquée par la vulnérabilité, les inégalités criantes, les attaques virulentes contre le concept de genre et les limites évidentes de la planète en matière de ressources, l’article souligne l’importance de l’engagement éducatif dans la politique du genre, les nombreuses formes que prennent ces liens et le potentiel de formation d’alliances. Afin de fournir un soutien solide au développement humain et à l’équité, tandis que ces valeurs sont remises en cause, il importe d’accorder une attention particulière aux informations tirées des données sur le genre, le sexe, la sexualité et l’intersectionnalité, à leurs cadres conceptuels, combinés à une compréhension empathique et à une pratique réflexive en vue de favoriser la solidarité.

## Introduction

This article considers how ideas about gender and education have been discussed, contested or overlooked in 70 years of work in the field of international and comparative education. It focuses on articles published in the *International Review of Education (IRE)* between 1955 and 2024, identifying contributions by women scholars and broader discussions of education and gender issues for differently situated women, girls, boys and men. It also reviews the research in this Journal on sex, sexuality and intersectionality.

In 2014, Nelly Stromquist published a detailed, reflective analysis of the work of Paulo Freire from a gender perspective. She wrote: “Our future will be always stronger when we recognise that knowledge is cumulative and thus a collective process” (Stromquist 2014, p. 556). Bolstering a history of cumulative scholarship requires examining how collective relationships are formed, supported and sustained. It also demands acknowledging challenges to these processes, considering forms of erasure or rejection, and reflecting on how debates and discussions deepen understanding. In preparing this article, looking at the work published in *IRE* that has contributed to discussions of gender in international and comparative education, I combined close analysis of contributions by female scholars and writers on women’s education and gender, with reflection on the intellectual themes that framed this work. I examined authorship, themes, locales and contexts. I also reflected on some overlooked themes, and sketched out a description of the approach to cumulative and collective scholarship that appears to be forming.

In this article, the argument proceeds in three parts. The first part presents my methodology and distils and periodises the main themes emerging between 1955 and 2024 in two groups of articles published in *IRE* – those written by women, and those addressing education and gender, sex, sexuality and intersectionality. I drew on techniques from systematic evidence reviews (Gough et al. 2017) to identify articles for analysis: selecting a sample of documents, screening for inclusion and exclusion, and mapping key themes. In this section, I note and periodise the changing gender composition of the authors*,* the contexts of gender studies and the main themes reviewed.

Even though I counted documents manually, without the benefit of the computational techniques and topic modelling used by Patricia Bromley and Daniel Scott Smith (2024) in their study of similar themes in two related journals over a shorter time period, some of our findings are similar. My analysis of 70 years of scholarship in *IRE* on gender, sex, sexuality and intersectionality reveals that while not all contributions on these themes were written by women, they represent the largest proportion of contributors to this discussion, confirming the findings of Bromley and Smith (2024). In the discussion, I reflect on potential reasons for this, engaging with Bromley and Smith’s interpretations about world society.

There is considerable debate about how evidence reviews in education have been and should be conducted (Wyse et al. 2025). The process of identifying the trends mapped in this article drew on insights from these discussions regarding rigour, transparency and reflexivity. However, this was not a systematic review; rather, it drew methodologically on approaches to intellectual history in comparative education concerned with periodisation, discourses and networks associated with the development of ideas (Moyn and Sartori 2013; Assie-Lumumba and Sutton 2004; Cowen 2020).

The second part of the discussion reflects on the broader scholarship in international and comparative education on gender, sex, sexuality and intersectionality, drawing on some commentaries on the field (Kelly 1990; Assie-Lumumba and Sutton 2004; Stromquist 2005; Fennell and Arnot 2008; Unterhalter 2014; Tamale 2020; Unterhalter 2023; Murphy-Graham 2024; Vanner 2025). It considers some of the major social movements concerned with gender and education over the period these review authors covered. In this section, I examine where work published in international and comparative education and contributions to *IRE* respond to these trends and where there are silences.

In the third part of the article, I explore the implications of this body of scholarship in relation to current conceptualisations and preoccupations around gender and education in 2024 and 2025 (e.g. Kamlongera 2025; Murphy-Graham 2024; Vanner 2025; Unterhalter 2025). I also examine their antecedents and the directions of thinking that appear to be emerging. Finally, I attempt to answer the question of whether or not a cumulative and collective scholarship has emerged.

## Seven decades of scholarship on gender and education in *IRE*

### Selection of articles for review

*IRE* was relaunched in 1955 into a field of international and comparative education marked by particular gender dynamics. The leading scholars, based at prestigious universities in the Global North, were virtually all men. Some notable exceptions to this trend were Margaret Read, who was working at the Institute of Education in London; Muriel Horrell, who was based at the Institute of Race Relations in South Africa; and Padmini Sengupta, who was writing in India across many transnational networks (Bagchi 2019; Unterhalter and Kadiwal 2022; Krige 2024). However, the work of these women has not been prominent, nor has the presence of large numbers of women in many countries working for decades in education as teachers, administrators, political activists, theorists and researchers, contributing to reshaping political, economic and cultural landscapes. While ideas and organisations concerned with the social and cultural construction of relationships linking education, sex, sexuality and intersectionality with other social divisions has spanned centuries, the term *gender* was only used to describe these relationships from the 1970s onwards (Stromquist 2005; Unterhalter 2014).

**Fig. 1:**
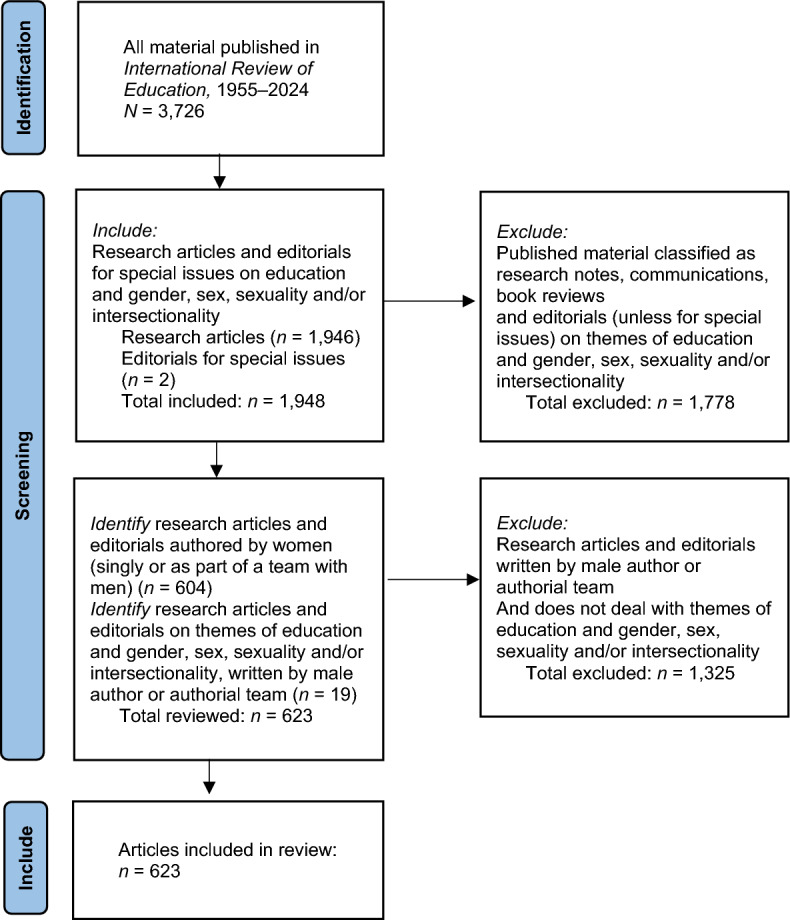
Process for selecting articles for review

To rigorously investigate these patterns in *IRE*, I developed an approach to identify relevant research articles written by women, either as single scholars or as part of teams working with male authors. All articles which dealt with themes of gender, sex, sexuality or intersectionality were included in the review, noting the gender of authors. Articles were excluded from the review if they were not classified as research articles by the Journal editor, if they were written only by male scholars, and if they did not address themes of gender, sex, sexuality or intersectionality. The flowchart in Fig. 1 details this process.

### Gender representation in authorship

In the first decade after the relaunch of *IRE*, of the 266 research articles published in the Journal, 14 were written by women, but only one dealt with the range of topics we now associate with work on gender and education (see Table 1). No male authorial team wrote on these issues during this period. The only article that examined some of these themes was published in 1956. Mary Green, principal of one of the first comprehensive schools in England,^1^ reflected on her experience setting up a secondary school for girls, as a female head teacher working with a team of mostly female teachers (Green 1956). She discussed fostering an inclusive ethos among staff and pupils, developing a strategy to guide the school in values based on religion and hard work, and establishing a strong organisational structure (ibid., pp. 422–423). However, gender as a concept did not feature in her discussion.

**Table 1:** Research articles published in *IRE* by sex of author and main topic

Decade of publication	Total number of articles published	Number of articles with female authors (% of all articles)	Number of articles addressing gender, sex, sexuality and/or intersectionality (% of all articles)	Number of articles by male authorial teams addressing gender, sex, sexuality and/or intersectionality (% of all articles)	Total number of articles for decade included in review
1955–1964	266	14 (5.3)	1 (0.4)	0	14
1965–1974	212	14 (6.6)	5 (2.4)	1 (0.5)	15
1975–1984	242	35 (14.5)	6 (2.5)	1 (0.4)	36
1985–1995	275	50 (18.2)	18 (6.5)	3 (1.1)	53
1995–2004	286*	107 (37.4)	31 (10.8)	3 (1.1)	110*
2005–2014	326*	177 (54.3)	50 (15.3)	10 (3.1)	187*
2015–2024	341	207 (60.7)	20 (5.9)	1 (0.3)	208
Total	1948	604 (31)	131 (6.7)	19 (0.98)	623

This table was compiled from a manual count of all the articles published in *IRE*, 1955–2024, checking each author’s name and reviewing each article’s main focus. The Supplementary Online Appendix (hyperlink provided in footnote 2) lists all the articles identified for discussion dealing with gender, sex, sexuality and/or intersectionality.

*Total includes one editorial introduction to a special issue on gender.

The absence of gender in Green’s article was not unique. As Table 1 indicates, a limited number of women wrote for *IRE* in the early decades, and discussion of gender was muted. The number of female authors grew significantly from the mid-1980s, as did the number of articles dealing with themes of gender, sex, sexuality and intersectionality, although there has been a marked decline in this focus in the most recent decade (2015–2024). Interestingly, as the number of female writers for the Journal increased, the attention given to gender, women’s education, sex and sexuality declined somewhat relative to its profile in earlier decades.

Over 70 years, discussion of these themes has reflected a wide range of concerns, moving from the personal and professional, as Green (1956) initially documented, to include studies commenting on communities, cultures, countries and the global landscape. Varied perspectives and disciplines have also been deployed, including anthropology, sociology, philosophy, psychology, politics, law, and women’s studies and planning. The overarching approach is both critical and exploratory across various fields of education studies, although a significant body of work has documented gender-related trends through surveys and quantitative data.

### Review process: inclusion and exclusion of articles

I conducted an audit of articles published in *IRE* between 1955 and 2024, as set out in Table 1, including only those that could be loosely classified as research-based. The articles included in the count had not been classified by the editors as editorials, communications, notes, research notes or book reviews. They were sometimes described as “main” or “original” articles. In some years, substantial introductory articles were included; however, editorial or introductory articles were generally excluded from my count. All audited articles were based on some form of academic inquiry, such as reporting on empirical studies, analysing conceptual debates or describing the work of education systems or institutions. Each article was examined to identify whether the author was female or male, and whether the themes discussed were concerned with gender, sex, sexuality and/or intersectionality.

In 2002, Knut Schwippert surveyed 46 years of work published in *IRE*, commenting on the gender of authors, country of origin and range of topics (Schwippert 2002). Schwippert’s survey was conducted before the internet provided extensive information on academic profiles, so my audit was more comprehensive than his. Nonetheless, he noted an increasing proportion of female authors towards the end of the 20th century (Schwippert 2002). Patricia Bromley and Daniel Scott Smith (2024) used computational analysis and hand coding of 2,517 articles published in two comparative education journals – *Compare* and *Comparative Education Review* – from their founding to 2010. They found few women noted as first authors before the 1980s, but a steep rise in this proportion to reach parity by 2016.

During my own audit, when an author’s name was not evident from the format in the Journal – for example, because only initials were provided, or because a name could be given either to a man or a woman – I used the Google search engine to investigate the individual. Notes on contributors to *IRE* were included in some issues, but this was not a consistent practice. Articles that were written by a woman, or co-written by a man and a woman, were included.

All articles were initially scanned by title to see whether they included themes concerned with gender, sex, sexuality or intersectionality. Any article that appeared to address these topics was read to assess its depth of treatment. Where this discussion was a key part of the analysis, the article was included in the summary of themes, as presented in Table 2.^2^ If the focus was on education equality or youth exclusion, for example, but there was only a cursory noting of gender differences and no discussion of implications, the article was not included. Examples of excluded articles are Robert Havighurst and Aparecida Gouveia’s (1966) consideration of attitudes to tradition and modernity among students in Brazil, which drew on a survey with male and female respondents but did not discuss gender perspectives, and William Taylor’s (1968) analysis of numbers of men and women enrolled in teacher education in England, which also did not reflect on this pattern. For many writers published in *IRE*, particularly in the early decades, gender was not a category of note.

**Table 2:** Research articles published in *IRE* (1955–2024) addressing education and gender, sex, sexuality and/or intersectionality by regional focus and decade of publication

Regional focus	Total number of articles*	1950s	1960s	1970s	1980s	1990s	2000s	2010s	2020s
Global or cross regional	35	0	0	3	1	6	14	9	2
Sub-Saharan Africa	32	0	0	1	3	7	7	12	2
Latin America and the Caribbean	8	0	0	0	1	4	2	1	0
Europe and North America	23	1	0	4	1	4	8	3	2
North Africa and West Asia	15	0	0	1	2	1	3	7	1
Central and South Asia	11	0	0	0	2	4	3	1	1
East and Southeast Asia	10	0	0	0	3	2	3	1	1
Oceania	4	0	0	1	3	0	0	0	0
Total	138	1	0	10	16	28	40	34	9

*The number of articles categorised by region is larger than the total number of articles on gender themes noted in Table 1 because some articles dealt with two countries or regions.

### Author identity and article theme

My audit reveals that, since 1955, 31 per cent of original articles published were written or co-written by women, although this proportion has changed markedly over the decades – from 5.3 per cent in 1955–1965 to 60.7 per cent in 2014–2025 (see Table 1). Across the seven decades, 6.7 per cent of articles addressed themes concerned with gender, sex, sexuality or intersectionality. While these themes had minimal presence in the early decades, they featured in 15.3 per cent of articles in 2005–2014, when the largest volume of studies on these topics was published.

In the first decade after the relaunch of *IRE*, and throughout the 1960s and 1970s – a period when women’s movements in a number of countries led to gender and women’s studies being included in academic discussion (Bowles and Klein 1980; Waylen et al. 2013; Hale 2022) – there was a limited presence of women writers in the Journal, and scant treatment of gender issues. This started to slowly shift from the mid-1990s. In the 21st century, of the 666 research articles and one editorial published in the two decades since 2005, 384 were by women writers, a marked change from the early period.

We thus observe a shift from women’s relative absence as contributors documenting and shaping educational discourse in the early decades of *IRE* to their significant presence in the current century. However, in the most recent period (2005–2024), while 70 articles addressed education and gender, sex, sexuality and/or intersections with other forms of social division or belonging, the number of articles on these themes has dropped markedly in the most recent decade (2015–2024) (see Table 1). An area for further investigation is why, despite the large numbers of women working in education throughout the 20th century in most of the countries covered by the Journal, so few were published as scholars in this field.

The identity of authors is intertwined with their areas of focus and the context from which they write. We can chart an arc from minimal to more substantive treatment of themes concerned with gender and women’s education. Table 1 reveals that not all women contributing to *IRE* focused on ideas about gender, while a small number of men did. However, over the 20th century, gender was not a mainstream concern in the Journal. In the 1950s, issues of social disadvantage, sometimes identified in terms of class or family relationships, were seen as major divisions (e.g. Isambert 1955; Wall 1955). From the 1960s, concerns with national planning and different formations of culture became the most common ways to frame educational experiences and aspirations (e.g. Husén 1963; Biobaku 1967).

In 2002, two articles commissioned for a special issue on the history of the Journal noted emerging themes, but did not single out gender. Joseph Zajda (2002) discussed changing approaches to addressing inequalities in education, without mentioning gender. Birgit Brock Utne (2002), in reviewing discussions in the Journal on education and development from 1931 to 2001, focused on three long-standing areas – nationalism and internationalism, language of instruction and aid policies. It appears, from these reviews and the manual count in Table 1, that work on gender only started to have a higher profile in the Journal after 2000.

### United Nations and regional influences

This finding on limited attention in *IRE* articles on gender in education is somewhat surprising, given that the Journal is affiliated with the United Nations Educational, Scientific and Cultural Organization (UNESCO). As Rosie Peppin Vaughan (2010) notes, the principle of educational equality was built into UNESCO’s constitution and is promoted to Member States through international conferences, reports and surveys. From 1977, in endorsing the resolutions associated with the United Nations (UN) Decade for Women (1975–1985), building on discussions conducted during the World Conference on Women held in Mexico City in 1975, UNESCO (1977) adopted the “Thinking Ahead” project (1977–1982). This project emphasised overcoming gender inequalities in education, although it mainly focused on equal access to formal schooling (Peppin Vaughan 2010; Leproni and Azara 2025).

The pattern of discussion in *IRE*, and the history of UNESCO’s engagement with gender equality in education, provides an interesting alternative perspective on gender and education discourse published in other settings. In an earlier reading of global policy literature on gender and education (Unterhalter 2014, 2008), I found a 1993 World Bank publication on women and girls’ education (King and Hill 1993) to be pivotal; it identified this investment as the most significant that governments and societies could make. Despite this, my current audit of articles published in *IRE* shows that while the importance of girls’ education was noted in some studies in the 1990s (see Table 3), it was not given the prominence the World Bank was promoting. In contrast, UNESCO’s interest in women’s education issues from the 1970s, most notably literacy, resonates with themes that *IRE* was publishing on.

**Table 3:** Research articles published in *IRE* (1955–2024) addressing education and gender, sex, sexuality and/or intersectionality by thematic focus

Thematic focus	Number of articles	Decade with most attention to theme and number of articles published
Conceptual/theoretical	19	2000–2009 (6)
Adult women’s learning	33	2000–2009 (12)
Forms of discrimination against girls in accessing and participating in school	29	2010–2020 (11)
Gender and teaching	8	1980–1989 (3); 2000–2019 (3)
Curriculum content (including sex education)	9	1990–1999 (4); 2000–2009 (4)
Gender and policy, administration, planning and management	16	2000–2009 (5)
Masculinities	1	2000–2009 (1)
Outcomes of girls in school (e.g. delayed marriage, labour market access)	7	2000–2009 (3)
Gender and higher education	8	2000–2009 (4)
Gender and social and emotional learning at school	2	2010–2019 (2)
Total	131	

Some dynamics about how and why gender has been emphasised by different UN organisations emerge from Table 2, which summarises regional clusters associated with different decades of the work of *IRE*. Of the 131 articles published between 1955 and 2024 (Supplementary Online Appendix), which I identified as addressing gender, sex, sexuality and/or intersectionality, the largest grouping discusses global and cross-regional features of these issues. Studies of sub-Saharan Africa, Europe and North America form the next largest grouping. There is a striking underrepresentation of studies from East and Southeast Asia, despite the considerable political and economic significance of these regions over this period. Populous regions, such as Latin America and South and Central Asia, are also significantly underrepresented. A group of studies on West Asia and North Africa were predominantly published during the decade of the Arab Spring (2010s) and the devastating wars in many countries of that region, but little of the published research on gender deals with these themes. Finally, a small number of articles on Oceania were mainly published after the World Council of Comparative Education Societies (WCCES) convened in Sydney in 1996.

Table 2 reveals that the Journal has, to an extent, reflected some of the concerns of UN agencies. Peppin Vaughan (2010) notes that after the 1975 Mexico World Conference on Women, mentioned above, UNESCO began focusing on substantive gender equality – not only in formal access to education but seeking to change the structures of inequality. The Director‐General was also required to submit a report to each General Conference on how UNESCO’s recent activities had contributed to improving the status of women. The 1977 medium‐term plan, *Thinking Ahead* (UNESCO 1977), prioritised women’s ability to exercise their education rights, not just the formal conferring of those rights. Furthermore, as gender difference came to be analysed as politically and socially constructed, a number of articles appeared in *IRE* addressing obstacles to equality in women and girls’ access to education. These initiatives were amplified in the 1990s, when UNESCO, the United Nations Children’s Fund (UNICEF) and the World Bank all began to focus on expanding girls’ access to school, although their approaches were different (Peppin Vaughan 2010; Unterhalter 2008).

The growing emphasis of these international organisations coincided with an increased number of globally focused articles on gender published in *IRE*. This included a larger presence of work on sub-Saharan Africa, Latin America and South Asia, which were all regions of focus for these organisations in thinking about education and international development. However, the largest waves of scholarship published in *IRE* discussing global trends and gender themes came from 2000, almost a decade after the World Bank volume (King and Hill 1993). More work on sub-Saharan Africa was published in the 2010s, when the number of scholarly works on South Asia and Latin America declined. The distribution of articles on Europe and North America comprised small subsets in all decades until the 2000s. In this decade, a group of articles was published, dealing with themes of “bending” gender, sexuality, migration and work. While gender, education, training and work were addressed in research on all regions (see Table 3), discussion of gender as a mutable category, and different ways of “performing” gender and questioning binaries, has only been the focus of articles on Western Europe and North America (e.g. Davidson 2009; Knotts 2009; Feuerverger 2011). Similarly, themes of migration and intersectional identities have been more prominently associated with work from these regions.

Given the Journal’s concern with lifelong learning from 2000, it is not surprising that the largest group of articles addressing themes of gender, sex, sexuality and intersectionality focuses on women’s experiences of learning in literacy classes, professional education and other work settings. The second largest group of articles concerns discrimination against girls in accessing, progressing through and completing school. This became a major policy focus of governments and UN agencies in the wake of the Millennium Development Goals (MDGs) and the Dakar Framework for Action on Education for All from 2000 (Unterhalter 2008; Monkman and Hoffman 2013). Some articles in this group address family relationships (e.g. Assy 2003; de la Caba Collado and Bartau Rojas 2010) and processes in schools (e.g. Chowdhury et al. 2003; Börkan et al. 2015). It is striking that relatively few articles address specific interventions to promote gender equality, for example, with regard to curriculum content or support for teachers. However, research on policy, planning, management and administration is a distinctive category, with 16 articles published, the majority in the decade after the launch of the MDGs (UN 2000) and the Dakar Framework for Action (WEF 2000).

Gender and higher education appears as a less visible theme in this audit. Among the articles published in this area, there is significant focus on Iran, a country that experienced substantial growth in the number of women enrolling in higher education from the late 1990s (e.g. Shavarini 2005; Mehran 2009). However, few articles remarked on this trend elsewhere in the world. Two articles published in the first decade of the 21st century linked gender identities with negative social and emotional experiences of school (Feuerverger 2011; Crossouard and Dunne 2015). Their presence signals a striking gap in this body of published literature with regard to school-related and gender-based violence, on which no articles were published, despite a rising interest in the issue in the wake of the HIV/AIDS pandemic beginning in the 1980s (Dunne et al. 2006; Parkes 2015), and later linked with wider concerns about violence against women (Parkes et al. 2016; van Daalen et al. 2022). Intersectional forms of discrimination associated with, for example, disability, race, ethnicity or contexts of conflict, are also striking in their absence from this corpus of published work.

### Men and women writing on gender and education

A final issue for consideration, on the basis of this audit, is why gender was not a category of interest for a large proportion of the men who worked in education studies over the 70 years under review – and relatedly, which women did and did not engage with these issues. Men’s involvement in work on gender equality has received some scholarly attention (e.g. Hearn 2011; Sweetman 2013), but very little discussion in research about research. Similarly, why some female scholars do not engage with issues of gender has been less documented than why some do. Further investigation is needed on both these points.

From my scan of titles and topics in articles published in *IRE*, it appears that for most authors, both men and women, gender disappeared into larger areas of focus associated with cultures, learning sites and education systems. Some women writers who discussed gender and education were developing an explicit feminist politics of scholarship (e.g. Acker 1987; Blackmore 1997; Stromquist 2014; Smythe 2022), while others were engaged with theory, method, policy or practice, where gender was a major theme for analysis. Few of the men who wrote on gender themes engaged with feminism.^3^ Table 4 distils and categorises male authors’ perspectives on these issues.

**Table 4:** Research articles published in *IRE* (1955–2024) written by men on education and gender, sex, sexuality and/or intersectionality by thematic focus

Thematic focus and number of articles published on this theme (derived from Table 3)	Articles co-written by men and women addressing education and gender, sex, sexuality and/or intersectionality	Single-authored articles by men addressing education and gender, sex, sexuality and/or intersectionality
Conceptual/methodological (19 articles)	2	2
Learning outcomes (7)	4	2
Access to schooling; participation and dropout (29)	15	5
Curriculum (9)	2	2
Adult women and lifelong learning (33)	3	4
Higher education (8)	2	1
Teachers (8)	2	1
Masculinities (1)		1
Policy and planning (16)	3	1
Total	33	19

The largest grouping of articles on gender themes published over seven decades (Table 4) focuses on women, gender and aspects of lifelong learning. Of these 33 articles, only seven involved male authors. This contrasts with the second largest grouping of articles on these themes, which highlights the obstacles to girls accessing, attending and participating in school. Of these 29 articles, 20 were written with the involvement of male scholars. This may be linked to this area being a major focus for UN organisations and statistical documentation. In contrast, women’s learning experiences were frequently documented in the Journal using qualitative methods, sometimes reporting on small-scale initiatives, often undertaken by female scholar practitioners (e.g. Mwiria 1993; Warkineh et al. 2018). Table 4 also highlights the limited engagement of male scholars in the discussion of conceptual, methodological, policy and planning issues associated with gender, sex, sexuality and intersectionality. The reasons for this require further investigation.

### The broader field of inquiry

The audit raises a number of questions about who the major interlocutors (e.g. political activists, theorists, policymakers, researcher/practitioners) were for the scholars published in *IRE*. Bromley and Smith (2024), in their analysis using neoinstitutional theory and computational techniques of matching and clustering in two comparative education journals, found that as the status of women expanded in liberal world culture, female authors, and research on feminism, gender, sex and sexuality, gained legitimacy and visibility in academic journals. While this provides useful framing, it does not help explain the pattern of topics both addressed and not considered in *IRE*.

Reflecting on the contextual concerns noted in Table 2, the themes documented in Table 3 and the identities of authorial teams highlighted in Table 4, it is clear that this published work is not the outcome of a single or homogenous group of scholars. Those who published work that was conceptual and methodological were in dialogue with other theorisations about how gender could be understood in relation to other social divisions (e.g. Blackmore 1997; Acker 1987; Smythe 2022), the historical formations of these ideas (e.g. Martin 1990; Megahed and Lack 2011), how gender inequalities might be undone (e.g. Stromquist and Fischman 2009) and how education rights, equalities and care connect with analysis of gender (e.g. Toh 1997; Lohrenscheit 2002). A feature of this work is a concern to describe and contextualise gender relationships in or through education, often linking them with forms of inequality, injustice, hierarchy and sometimes violence. I see this discussion as a kind of connection, or intermingling, of gender scholarship with other forms of theorising in education about ethical processes, guiding ideas and the contexts that shape them. Thus, the work published in *IRE* resonates with the way this scholarship has been unfolding more generally in writing about women, gender and education (e.g. Stromquist 2005; Unterhalter 2014; Kwachou 2023; Roy 2024).

A second group of interlocutors is more linked to practice – particularly in policy development and implementation, and in identifying forms of gender discrimination in order to ameliorate or prevent it across all phases of education. The desire to develop feminist pedagogies and gender-responsive practices in management, assessment, research and learning support is a widespread theme in work on women’s rights in and through education, in analyses of the experiences of both girls and boys in schools, and in efforts to reduce violence and build safe learning environments (see e.g. Shrewsbury 1993; Elwood 2005; Chapin and Warne 2020; Kataeva et al. 2024). Two prominent clusters of articles published in *IRE* engage with practice. The first cluster uses quantitative methods to analyse the different experiences of and outcomes for girls and boys in schooling (e.g. Gorman and Politt 1992; Kim 2018). The second body of scholarship reflects on how to conduct gender-responsive practices, generally linked to lifelong learning (e.g. Toh 1997), policy (e.g. Mannathoko 1999), planning (e.g. Guarino and Tanner 2012) and pedagogy (e.g. Arnot 2006).

A third type of interlocutor, prominent in literature on gender and education, concerns debates about reflexive accounts of educational experiences and how these are researched (e.g. Rose 1997; Reay 1996). Documentation of the uneven processes of inclusion and exclusion in studying and working across all phases of education has been both a major theoretical orientation in this scholarship and a key way in which data have been assembled and critiqued, and insights accumulated and reviewed (e.g. Kenway and McLeod 2004). In *IRE* articles that have contributed to this gendered perspective on reflexivity, learners were situated in a variety of settings – for example, in a literacy class (e.g. Witenstein and Iyengar 2021), in vocational education (e.g. Munro et al. 2000) or in academia (e.g. Hickling Hudson 2006; Brock-Utne 2018; Halvorsen 2018). For all these differently positioned writers, documenting and reflecting on gender, identity and learning was a key form of exploration and discussion. A distinctive, authorial voice emerged, associated with scholarship about gender and its complex interplay with other relationships, structures, artefacts and ideas. Some of these writers sought to develop a gender perspective in more conceptual pieces, linking their discussion to understanding forms of power and associating it with other emancipatory ideas for education, such as empowerment and social justice (e.g. Stromquist 2014; Smythe 2022).

A key question is whether the small shifts and incremental accumulation of insights, documented over the decades, both in *IRE* and in the wider field of inquiry, have strengthened the field of investigation regarding gender and women’s rights in and through education. A related question is whether this approach, evident through the themes addressed in the Journal, has risked leaving in place forms of patriarchy and misogyny in some education systems, with limited advice on how to challenge and dismantle these structures. One way to answer these questions is to consider how the authors, in researching and writing on gender, positioned their work in relation to organisations and social movements for change associated with the different decades under review. These social movements were a key context for literature on gender and international and comparative education, as a number of studies note (Stromquist 2005; Unterhalter 2014). In the next section, I examine how the published work linking gender and education in *IRE* did and did not resonate with major social movements advocating for women’s rights, gender equality and education from the 1950s to the 2020s.

## Social movements, gender and education: scholarly engagements and disconnections

For the first four decades of *IRE*, although women’s movements demanding gender equality in education were a significant feature in the politics of many countries, in both the Global North and South (Ferree and Mueller 2004; Pelak et al. 2006; Antrobus 2008; Peppin Vaughan 2019), and while gender in education was being addressed in scholarship on education policy, literacy and development, the Journal gave little prominence to these ideas or processes. A handful of articles, published between 1955 and 1995, signalled these profound changes. However, with the exception of Sandra Acker’s work, discussed below (Acker 1987), these articles were infrequently cited and engaged with by other authors contributing to *IRE*.

An example is Maureen Woodhall’s article, published in 1973, on the importance of reviewing human capital theory when thinking about investment in women’s education. She noted the liberal feminism of the 1960s in Europe and North America, and presented data on the earnings and economic contribution of women with higher levels of education (Woodhall 1973). However, a Google Scholar citation report I consulted during my research reveals that Woodhall’s article was only cited by a small number of scholars publishing in education journals, and only one other contributor to *IRE.*^4^ Aruna Roy (1980), writing on women’s activism in India, documented local initiatives to appoint female teachers to jobs in rural settings to support the communities they knew well. Her study can be placed in the social history of grassroots activism in India from the 1970s, which Roy’s memoir (Roy 2024) recounts, and other historians have described, with huge significance for political, social and educational initiatives (e.g. Basu 1995; Antrobus 2008). Roy’s article for *IRE* has not been cited at all by other contributors to this or other education journals.^5^ Thus, it appears that, at least until the mid-1990s, interlocutors for the work on gender published in *IRE* mainly stood outside the field of comparative and international education.

There is one exception to this trend. In 1987, Sandra Acker’s article “Feminist theory and the study of gender and education” described three major currents in Western feminist scholarship—liberal, radical and socialist (Acker 1987). This segmentation became a standard way in which the scholarly field of women’s studies and education, and research into gender and education, came to be organised for at least two decades, shaping reflections on theory, methods, policy and practice (see e.g. Thompson 2003; Arnot and Weiler 2005). The emerging academic discourse on gender, education and international development was very much in dialogue with these ideas (e.g. Heward and Bunwaree 1999; Unterhalter 2005; Kwachou 2023). These academic and practice interlocutors were sporadically published in *IRE* and other education journals. Acker’s article is historically significant, not only for its extensive citation^6^ and the engagement it generated, but also because it marks a point after which the Journal started to give more space to work on gender and feminist themes (see Table 1). However, as Table 1 shows, this was always a small component of the work published.

Following Acker’s seminal article, other conceptual pieces on gender, feminist theory and education were published by *IRE* in subsequent decades. However, on the basis of their citation history, these articles appear to have had less far-reaching influence on how gender and education appeared in academic scholarship, with implications for visibility in course content in universities.^7^ These articles, nonetheless, contributed to the development of particular positions on political mobilisation and women’s literacy, and it is here that I think their most significant impact may be traced. In a special issue published in 2011, Carolyn Medel-Añonuevo and Anna Bernhardt (2011) mapped how the 1995 Beijing World Conference on Women brought gender and women’s literacy to the forefront in the series of International Conferences on Adult Education (CONFINTEA). Shirley Walters and Linda Cooper (2011), contributing to the same CONFINTEA VI follow-up special issue, argued that understanding gender and intersectionality is intricately connected to analysing power struggles over learning linked to agendas about skills and work.

The link between feminist activism and literacy work inspired by Paulo Freire was a key theme for Nelly Stromquist (2014). Suzanne Smythe (2022), in a highly critical reading of the assumptions of progress and techno-rationalism in lifelong learning, asserts the need to draw instead on Frantz Fanon and Sylvia Wynter for ideas about the “hybrid human”, in which gender is understood through speculative, destabilising enquiry, and through “storying” practices in a world that is unsettled and unsettling. Thus, the feminist politics that have come to be associated with lifelong learning in *IRE* do not have only one complexion; rather, they raise questions about confronting and changing injustice. This plasticity with regard to key ideas associated with feminist work in education, and scholarship on gender—for example, empowerment, reflexivity, identities and intersectionality—has also been a feature of the wider field of enquiry.

These ideas concerning feminism and lifelong learning appear to be the most discernible group of interlocutors for writers in *IRE* concerned with gender and social change. They may be contrasted with other groups of social activists concerned with gender, who are less visible in the articles the Journal has published. From the 1990s, social movements articulating and organising around gender identities, including sexual orientation, began to be documented in many countries (Basu 1995; Meyer et al. 2002). This theme was taken up in some of the articles in the 2009 special issue on “undoing” gender, co-edited by Nelly Stromquist and Gustavo Fishman (2009). For example, Samuel Davidson (2009) wrote about masculinities, “gender bending” and heteronormativities based on narrative research with teenage boys in the United States. In a later special issue featuring selected papers from the XIV World Congress of Comparative Education Societies held in Istanbul in 2010, Grace Feuerverger (2011) wrote about experiences of trauma linked to refugee learners’ identities in a Toronto high school. However, this theme has not been prominent in the Journal, and the issue of intersectional identities linked to race has had very limited discussion, with Smythe (2022) highly critical of approaches that merely add Black or Indigenous experiences of gender or other social division, while retaining the main framing of analysis.

## A cumulative and collective scholarship?

This article has briefly examined how some social movements concerned with women’s rights and gender have been reflected in the articles published in *IRE* over 70 years, while others have been overlooked. It has also aimed to clarify whether scholarship on women’s education and gender represents a cumulative, collective contribution to broader social movements, or whether the Journal’s articles should be read as individual contributions—by scholars working alone or in organisations with some concern for gender, but little strategic oversight of the field’s development or for charting the directions of social change.

We can read this corpus of work in *IRE* in several ways. Although the Journal was slow to publish female scholars and to showcase research on gender and education, insights have accumulated, as shown by the audit presented in this article, and these insights resonate with broader scholarship. More research is needed to write a full intellectual history of the emergence of these ideas, including what prompted their articulation and how they were received. But it is clear that the Journal has contributed to a substantial body of conceptual and empirical work on gender and education in a wide range of settings. However, it is harder to judge whether there is a collective or solidaristic dynamic to this scholarship.

The years of 2024 and 2025 mark an inflection point of acute anxiety in gender and education. The contemporary moment is shaped by contradictory processes, with gender acting as a kind of *shibboleth* or form of distinction—sorting and characterising different kinds of educational initiatives. It is an age of marked attacks on practical and conceptual work on gender in education in a number of countries (Butler 2024; Reinhardt et al. 2024). These critiques have come from both conservative and authoritarian political orientations, concerned to preserve particular forms of the family or religious hierarchy, and from critical scholars who reject the ways in which a focus on women resonates with ideas of “white saviourism” (Zakaria 2021), or where support for trans identities is seen to question social policy decisions – which commentators consider require an appreciation of biology (Sullivan and Todd 2023). Statistics about gender and education are both celebrated and reviled (e.g. Bonfert and Wadhwa 2024; Grown 2025). These critiques are taking place under conditions of widening inequality and prominent authoritarian regimes, with gender often related to an intensification of vulnerabilities and injustices. Reports have been assembled in many countries of a widespread sense of insecurity. Despite access to higher incomes and better education and health provision, women and LGBTQ+ communities in many settings have linked this insecurity with forms of gender relationships (UNDP 2022; UNDP 2024). Additionally, the realities of planetary boundaries with regard to resources, and the inadequacies of current economic and social relationships, including the fragile multilateral order, to adequately mitigate threats and prevent greater insecurity is a matter of huge concern.

Ten years ago, Nelly Stromquist associated empowerment within the women’s movement with “profound gender emancipatory learning and sociocultural awareness” (Stromquist 2014, p. 556). But she also highlighted political insights into “the indispensable need to have clear and specific political goals, to seek allies, and to develop tools and strategies to foster as well as to engage in political action” (ibid.). The work on gender published in *IRE* helps to build context for this strategic action; however, its range is small. Many structures that maintain unequal power, gross violations of human rights and threats to whole populations have not been directly challenged or fundamentally changed. Hostile challenges to the work of international organisations on gender and other areas of human rights have also mounted over the period of writing this article.

Scholarly work is political in largely indirect ways. Whether research and analysis can support and sustain movements, practices and institutions capable of undoing the large injustices and inequalities of our time is an open question. In 2002, Robin Burns (2002) surveyed how articles in *IRE* over five decades had positioned education in relation to social change. She noted that authors generally viewed education as a conservative force, supporting processes for socialisation and economic development. It was only in times of prosperity that experimentation with equity was undertaken. In contrast, my current analysis suggests that writers engaging with gender, sex, sexuality, intersectionality and women’s rights have often stood in opposition to this view. Their work in the Journal is not conservative, and their voices have been raised both in times of economic growth and political and economic recession. Overall, while there are many facets to work on gender and education, there is also a general direction of travel that notes inequality and seeks change. While for some writers this is minimal, for others it is substantial and transformational.

This concern to both aim for and effect social change resonates with Stromquist’s invitation to build a cumulative scholarship. As we enter a new period of grave dangers and vulnerabilities, powerful authoritarian states, and attempts to weaken and discredit ideas about gender, education, rights, connection, solidarity and equality, we need the insights from this critical scholarship as never before. Educational engagements with the politics of gender, the many forms these connections take and their potential for alliance building – drawing on insights from data and empathetic understanding – can provide essential support for human development and equity. Knowing the history of these ideas and the people who shaped them can contribute to maintaining connections, and help retain a collective vision for profound change, seeking to build together more gender-just, equitable and humane relationships.

## Supplementary Information

Below is the link to the electronic supplementary material.

Supplementary file1 (DOCX 75 KB)

## Data Availability

Not applicable.

## Footnotes

1 In England, a comprehensive school refers to a non-selective school, which served all pupils of secondary school age in a certain area, regardless of their level of ability or aptitude. The term was first used in the 1940s and is still in use today.

2 A full list of articles reviewed for Table 2 is provided in the Supplementary Online Appendix, available at https://doi.org/10.1007/s11159-025-10193-y.

3 From my audit, this was only discussed in the article co-written by Gustavo Fischman with Nelly Stromquist (Stromquist and Fischman 2009)

4 Google Scholar (consulted 23 March 2025) reported 41 citations of this work. Only one was in *IRE*, and nine were in other education journals.

5 Google Scholar (consulted 7 February 2025) reported seven citations. None were in education journals. According to Springer, this article was cited twice in Web of Science (WoS) publications: https://link.springer.com/article/10.1007/BF01427501/metrics

6 Google Scholar (consulted 16 March 2025) reported that the article was cited by 406 authors.

7 Google Scholar searches (February 2025) indicated 402 citations of Acker (1987), 85 for Stromquist and Fischman (2009) and 92 for Stromquist (2014).
