# Supplementary material for: Cumulative, collective or conservative? A review of seven decades of writing about women, gender, sex, sexuality and intersectionality in international and comparative education
Source: Int Rev Educ. 2025 Nov 4;71(5-6):835–58. doi: 10.1007/s11159-025-10193-y (PMC12662869; doi:10.1007/s11159-025-10193-y)
Supplement: Supplementary file 1 — Supplementary file1 (DOCX 75 KB) [file 11159_2025_10193_MOESM1_ESM.docx]

Elaine Unterhalter

Supplementary Online Appendix for article

<https://doi.org/10.1007/s11159-025-10193-y> *Int Rev Educ* (2025)

Articles about women, gender, sex, sexuality, intersectionality

Key

| Conceptual/theoretical studies | Studies of curriculum content (Including sex education) | Studies of policy |
| --- | --- | --- |
| Studies of adult women learning and mobilising | Studies of gender and administration, planning and management | Higher education students |
| Studies of forms of discrimination against girls in school – access, participation, learning outcomes | Studies of masculinities | Social and emotional learning at school associated with gender |
| Studies of gender and teaching | Studies of the outcomes of girls in school (delayed marriage, labour market access etc) |  |

Chronological list of articles reviewed

| **Author(s)** | **Year** | **Article** | **Focus** |
| --- | --- | --- | --- |
| Mary Green | 1956 | Kidbrooke school: An English comprehensive school. *IRE, 2*(4), 419–431. <https://doi.org/10.1007/BF01421054> | Describes experiences setting up Kidbrook, approach as a woman head, and thoughts about women teachers |
| Evelyn Broomhead | 1973 | The education of women. *IRE,19*(1), 5–8.  <https://doi.org/10.1007/BF00597772> | Discusses continuing inequalities against women and forms of education trying to redress this |
| Maureen Woodhall | 1973 | Investment in women: A reappraisal of the concept of human capital. *IRE,* 19(1), 9–29.  <https://doi.org/10.1007/BF00597773> | assesses how human capital writing has neglected to consider investment in women |
| Marie Eliou | 1973 | Scolarisation et promotion feminines en Afrique francophone (Côte-d’Ivoire, Haute-Volta, Sénégal) [The education and advancement of women in Africa (Ivory Coast, Upper Volta, Senegal)]*. IRE, 19*(1), 30–46. <https://doi.org/10.1007/BF00597774> | Distribution of enrolments for girls at different levels |
| John Keeves | 1973 | Differences between the sexes in mathematics and science courses*. IRE, 19*(1), 47–63. <https://doi.org/10.1007/BF00597775> | Looks at IEA data and sex differences in opportunities to learn maths |
| Ingrid Fredriksson | 1973 | Sex roles and education*. IRE, 19*(1), 64–76. <https://doi.org/10.1007/BF00597776> | Sweden’s education policy and attempts to develop inter-relationship between sex roles |
| Henri Janne | 1975 | Educational needs of the 16–19 age group: A sociological perspective*. IRE, 21*(2), 127–148. <https://doi.org/10.1007/BF00598119> | Some discussion of how forms of patriarchal family and traditional images of gender affect educational participation; but minimal discussion |
| Suzanne Shafer | 1976 | The socialization of girls in the secondary schools of England and the two Germanies*. IRE, 22*(1), 5–23. <https://doi.org/10.1007/BF00597885> | Socialisation of girls and how they think about employment. Compares England, GDR and West Germany |
| Aruna Roy | 1980 | Schools and communities: An experience in rural India*. IRE, 26*(3), 369–378. <https://doi.org/10.1007/BF01427501> | Describes local activism to appoint teachers with knowledge of rural life in schools in India. Many of the teachers are women, draws out issues of their social activism |
| Lois Weis | 1983 | Inequality in Ghanaian secondary schools: Educational expansion, recruitment, and internal stratification*. IRE, 29*(1), 21–36. <https://doi.org/10.1007/BF00597558> | Looks at socio-economic background of male and female secondary school pupils. Some discussion of gender distribution |
| Celina Garcia | 1983 | Latin American traditions and perspectives*. IRE, 29*(3), 369–389. <https://doi.org/10.1007/BF00597980> | Need for peace education in Latin America to challenge the pathology of the violent man and traditional gender roles |
| Aletta Grisay | 1984 | Analyse des inégalités de rendement liées au sexe de l'élève dans l'enseignement primaire ivoirien [Analysis of gender-related performance inequalities in primary education in Côte d'Ivoire]*. IRE, 30*(1), 25–39. <https://doi.org/10.1007/BF00598016> | Girls’ reduced learning outcomes compared to boys in developing countries |
| Konai Thaman | 1987 | A Tongan teacher's story*. IRE, 33*(3), 277–281. <https://doi.org/10.1007/BF00615299> | Firsthand account of work as a teacher and activities of a village women’s committee |
| Sandra Acker | 1987 | Feminist theory and the study of gender and education*. IRE, 33*(4), 419–435. <https://doi.org/10.1007/BF00615157> | Gender theorising |
| Zakia Belhachmi | 1987 | The unfinished assignment: Educating Moroccan women for development*. IRE, 33*(4), 485–494. <https://doi.org/10.1007/BF00615161> | Linking education and women’s liberation and infrastructure |
| Renate Nestvogel | 1987 | Frauen, Bildung und Entwicklung*. IRE, 33*(4), 467–484. <https://doi.org/10.1007/BF00615160> | Links of issues around gender with environment and colonialism |
| Swarna Jayaweera | 1987 | Gender and access to education in Asia*. IRE, 33*(4), 455–466. <https://doi.org/10.1007/BF00615159> | Patriarchal social structures and gender constraints on enrolment |
| Milton M. Adams & Susan E. Kruppenbach | 1987 | Gender and access in the African school*. IRE, 33*(4), 437–453. <https://doi.org/10.1007/BF00615158> | Gender and patterns of enrolment |
| Karen Bellefleur Murray | 1988 | Profile of the new generation of teachers in the Turkish educational system*. IRE, 34*(1), 5–15. <https://doi.org/10.1007/BF00601915> | Discusses data on preferences for teaching; draws out some gender distribution, but minimal discussion |
| Jane Roland Martin | 1990 | Filling the gap: The goals of American education revised*. IRE, 36*(2), 145–157. <https://doi.org/10.1007/BF01874880> | How gender and issues about home and family devalued in aims of education discussions |
| Lynn Ilon & Peter Moock | 1991 | School attributes, household characteristics, and demand for schooling: A case study of rural Peru*. IRE, 37*(4), 429–451. <https://doi.org/10.1007/BF00597620> | Mothers’ education and attitudes to girls’ schooling |
| Kathleen S. Gorman & Ernesto Politt | 1992 | Gorman, K.S., Pollitt, E. School efficiency in rural Guatemala*. IRE, 38*(5), 519–534. <https://doi.org/10.1007/BF01100990> | Girls high dropout rate in data |
| M. Eugenia Dengo de Vargas | 1993 | The educator's point of view*. IRE, 39*(1–2), 19–24. <https://doi.org/10.1007/BF01102438> | Very minimal discussion of concern with women’s rights as part of expansion of education |
| O. J. Sikes, Jairo Palacio & Beverley Kerr | 1993 | Key non-controversial concepts of population education*. IRE, 39*(1–2), 31–36. <https://doi.org/10.1007/BF01102440> | Some discussion of sex education |
| Fama Hane Ba | 1993 | Femme et éducation: une équation déterminante pour le développement humain en Afrique [Women and education: A crucial equation for human development in Africa]*. IRE, 39*(1–2), 88–91. <https://doi.org/10.1007/BF01102446> | Social conditions and women’s education |
| George Muito | 1993 | Initiatives and resistances in English-speaking African countries*. IRE, 39*(1–2), 97–102. <https://doi.org/10.1007/BF01102448> | Minimal discussion of gender, but does discuss family planning education. (additional 8 articles in this issue that do the same) |
| Patrick C. F. Kwan | 1993 | Singaporean gifted adolescents under scrutiny: The gender factor*. IRE, 39*(3), 161–182. <https://doi.org/10.1007/BF01102400> | Gifted girls and ideas about femininity |
| Kilemi Mwiria | 1993 | Kenyan women adult literacy learners: Why their motivation is difficult to sustain*. IRE, 39*(3), 183–192. <https://doi.org/10.1007/BF01102401> | Women’s responsibilities making it hard to persist in literacy classes |
| Paola Belpassi | 1994 | Le conte africain: l’univers de l’oralité dans le système de l’enseignement [African storytelling: the world of oral tradition in the education system]*. IRE, 40(*3–5), 257–270. <https://doi.org/10.1007/BF01257780> | African storytelling and morals about gender relations |
| Maria Teresa Sirvent | 1994 | The politics of adjustment and lifelong education: The case of Argentina*. IRE, 40(*3–5), 195–207. <https://doi.org/10.1007/BF01257775> | Minimal comment on women and men registering for adult ed courses. Generally no discussion of gender |
| Carmel Borg, Jennifer Camilleri, Peter Mayo & Toni Xerri | 1995 | Malta’s National Curriculum: A critical analysis*. IRE, 41*(5), 337–356. <https://doi.org/10.1007/BF01103033> | Critiques Malta’s new curriculum for conservative attitudes to women |
| Lene Buchert | 1995 | The concept of Education for All: What has happened after Jomtien? *IRE, 41*(6), 537–549. <https://doi.org/10.1007/BF01263146> | Discusses EFA and distribution education enrolment and attainment by gender |
| Maria Luisa Canieso-Doronila | 1996 | 7. The Philippines [*Special section* Adult education – The legislative and policy environment]*. IRE, 42*(1–3), 109–129. <https://doi.org/10.1007/BF00597961> | Legislative environment for adult education. Some discussion of gender issues |
| Ila Patel | 1996 | 5. India*.* [*Special section* Adult education – The legislative and policy environment]*. IRE,* 42(1–3), 75–96. <https://doi.org/10.1007/BF00597959> | Some discussion of gender distribution of illiteracy |
| Geert Driessen and Hetty Dekkers | 1997 | Educational opportunities in the Netherlands: Policy, students’ performance and issues*. IRE, 43*(4), 299–315. <https://doi.org/10.1023/A:1003071705614> | Looks at student learning outcomes and exam performance. Notes how gender does not play a role |
| Chandra Gunawardena | 1997 | Problems of illiteracy in a literate developing society: Sri Lanka*. IRE, 43*(5–6), 595–609. <https://doi.org/10.1023/A:1003010726149> | Literacy and numeracy rates by gender in surveyed population |
| Helen Geissinger | 1997 | Girls' access to education in a developing country*. IRE, 43*(5–6), 423–438. <https://doi.org/10.1023/A:1003029820697> | Barriers preventing girls accessing education in Papua New Guinea |
| Jill Blackmore | 1997 | Level playing field? Feminist observations on global/local articulations of the re-gendering and restructuring of educational work*. IRE, 43*(5–6), 439–461. <https://doi.org/10.1023/A:1003038021606> | Continuing gender inequalities in education work – Australia, New Zealand, Sweden, Israel |
| Swee-Hin Toh & Virginia Floresca-Cawagas | 1997 | Towards a people-centred education: Possibilities and struggles in the Philippines*. IRE, 43*(5–6), 527–545. <https://doi.org/10.1023/A:1003050407493> | Discusses Freirean education movements and association with women’s empowerment |
| Ila Patel | 1998 | The contemporary women's movement and women's education in India*. IRE, 44*(2–3), 155–175. <https://doi.org/10.1023/A:1003125808644> | Feminism, education and women’s movement in India |
| Elie Ghanem | 1998 | Social movements in Brazil and their educational work*. IRE, 44*(2–3), 177–189. <https://doi.org/10.1023/A:1003130025482> | Women’s movements and education in Brazil |
| Lynn Ilon | 1998 | The effects of international economic trends on gender equity in schooling*. IRE, 44*(4), 335–356. <https://doi.org/10.1023/A:1003217807277> | Gender and schooling |
| Mehrotra, S. | 1998 | Education for All: Policy lessons from high-achieving countries*. IRE, 44*(5–6), 461–484. <https://doi.org/10.1023/A:1003433029696> | Expanding participation women in secondary education in selected countries |
| Samir R. Nath, Kathy Sylva & Janice Grimes | 1999 | Raising basic education levels in rural Bangladesh: The Impact of a non-formal education programme*. IRE, 45*(1), 5–26. <https://doi.org/10.1023/A:1003548518474> | Evaluation of a BRAC NFE intervention. Some data on girls and boys |
| Enrique Jacoby, Santiago Cueto & Ernesto Pollitt | 1999 | Determinants of school performance among Quechua children in the Peruvian Andes*. IRE, 45*(1), 27–43. <https://doi.org/10.1023/A:1003521804362> | Looking at mothers’ and fathers’ education as a variable in children’s attainment |
| Alexandros Kakavoulis & Joan Forrest | 1999 | Attitudes and values in sexual behaviour and sex education: A cross-cultural study among University students in Greece and Scotland*. IRE, 45*(2), 137–150. <https://doi.org/10.1023/A:1003688807427> | Survey of attitudes to gender identity and sexual relationships and experience of sex ed |
| Claudia Mitchell, Marilyn Blaeser, Barbara Chilangwa & Irene M. Maimbolwa-Sinyangwe | 1999 | Girls’ education in Zambia: Everyone's responsibility – A policy framework for participatory process*. IRE, 45*(5–6), 417–430. <https://doi.org/10.1023/A:1003854304335> | Promoting girls’ education – policy, practice etc |
| Changu Mannathoko | 1999 | Theoretical perspectives on gender in education: The case of Eastern and Southern Africa*. IRE, 45*(5–6), 445–460. <https://doi.org/10.1023/A:1003866707061> | Promoting girls’ education – policy, practice etc |
| Azzedine si Moussa & Frédéric Tupin | 1999 | Les systèmes éducatifs à la Réunion et l'île Maurice: quelle efficacité sociale? [The education systems in Réunion and Mauritius: How effective are they socially?] *IRE, 45*(5–6), 529–546. <https://doi.org/10.1023/A:1003851730260> | Looking at quality of ed and girls’ and boys’ experiences in Mauritius and Reunion |
| Rosemary Preston | 1999 | Critical approaches to lifelong education*. IRE, 45*(5–6), 561–574. <https://doi.org/10.1023/A:1003835209787> | Some general discussion of gender and inequality |
| Zellyne Jennings | 2000 | Functional literacy of young Guyanese adults*. IRE, 46*(1), 93–116. <https://doi.org/10.1023/A:1003926406978> | Some discussion of women with higher levels of functional literacy. But no intersectional discussion |
| Ali Arayıcı | 2000 | Les disparités d’alphabétisation et de scolarisation en Turquie [Disparities in literacy and schooling in Turkey]*. IRE, 46*(1), 117–146. https://doi.org/10.1023/A:1003934708795 | Women /men differences in literacy; no intersectional analysis |
| Kaori H. Okano | 2000 | Social justice and job distribution in Japan: Class, minority and gender*. IRE, 46*(6), 545–563. <https://doi.org/10.1023/A:1026574827232> | Discussion of how gender not considered in relation to vulnerabilities or equality in job support in Japan |
| Anne Munro, Lesley Holly & Helen Rainbird | 2000 | “My ladies aren’t interested in learning”: Managers, supervisors and the social context of learning*. IRE, 46*(6), 515–528. <https://doi.org/10.1023/A:1026526911303> | Discriminatory ideas about women workers in Uk |
| Knut Schwippert | 2002 | Forty-six years of *IRE*: A statistical and documentary survey*. IRE, 48*(1–2), 111–129. <https://doi.org/10.1023/A:1015646109796> | Increasing presence of women authors in *IRE* |
| Robin Joan Burns | 2002 | Education and social change: A Proactive or reactive role? *IRE, 48*(1–2), 21–45. <https://doi.org/10.1023/A:1015654311613> | Reviews some articles on women and gender in survey of *IRE.* Threats to gains on gender equality |
| Felisa Tibbitts | 2002 | Understanding what we do: Emerging models for human rights education*. IRE, 48*(3–4), 159–171. <https://doi.org/10.1023/A:1020338300881> | Some discussion of a human rights education model geared to women’s empowerment; intersectionality |
| Claudia Lohrenscheit | 2002 | International approaches in human rights education. *IRE, 48*(3–4), 173–185. <https://doi.org/10.1023/A:1020386216811> | Identifies learning for human rights linked with gender equality and empowerment; intersectionalities |
| Sabine Hornberg | 2002 | Human rights education as an integral part of general education*. IRE, 48*(3–4), 187–198. <https://doi.org/10.1023/A:1020330131832> | HRE and approaches to inequalities including gender |
| Nirmala Rao, Kai-Ming Cheng & Kirti Narain | 2003 | Primary schooling in China and India: Understanding how socio-contextual factors moderate the role of the state*. IRE, 49*(1–2), 153–176. <https://doi.org/10.1023/A:1022969922200> | Draws out different approaches to ed of girls in India and China |
| Edmond Paul Assy | 2003 | Dynamique socio-économique et crise familiale et éducative en Côte-d’Ivoire de 1960 à 1990 [Socio-economic dynamics and the family and educational crisis in Côte d’Ivoire from 1960 to 1990]*. IRE, 49*(5), 433–462. <https://doi.org/10.1023/A:1026340621603> | Instability and changes in family affecting education and social relations |
| A. Mushtaque R. Chowdhury, Samir R. Nath & Rasheda K. Choudhury | 2003 | Equity gains in Bangladesh primary education*. IRE, 49*(6), 601–619. <https://doi.org/10.1023/B:REVI.0000006929.59667.16> | Girls lagging behind boys in learning outcomes |
| James S. Page | 2004 | Peace education: Exploring some philosophical foundations*. IRE, 50*(1), 3–15. <https://doi.org/10.1023/B:REVI.0000018226.19305.6c> | Ethics of care and peace education |
| Mitra K. Shavarini | 2005 | The feminisation of Iranian higher education*. IRE*, *51*(4), 329–347. <https://doi.org/10.1007/s11159-005-7738-9> | Stresses voices of women |
| Solomon Sibiya& Linda van Rooyen | 2005 | Illiterates in South Africa: Who are they and what motivates them to participate in literacy campaigns? *IRE, 51*(5–6), 479–497. <https://doi.org/10.1007/s11159-005-2557-6> | Gender issues and participation in literacy. Empowerment and women’s literacy |
| Anne Hickling Hudson | 2006 | Cultural complexity, post-colonialism and educational change: Challenges for comparative educators*. IRE, 52*(1–2), 201–218. <https://doi.org/10.1007/s11159-005-5592-4> | Draws on autobiography to bring out intertwining race, gender, colonial relationships. Intersectional analysis |
| W. James Jacob | 2006 | Social justice in Chinese higher education: Regional issues of equity and access*. IRE, 52*(1–2), 149–169. <https://doi.org/10.1007/s11159-005-5613-3> | Gender gap in Chinese higher education |
| Madeleine Arnot | 2006 | Freedom’s children: A gender perspective on the education of the learner-citizen*. IRE, 52*(1–2), 67–87. <https://doi.org/10.1007/s1159-005-5611-5> | Gender and citizenship |
| Mary Khakoni Walingo | 2006 | The role of education in agricultural projects for food security and poverty reduction in Kenya*. IRE,* 52(3–4), 287–304. <https://doi.org/10.1007/s11159-006-0008-7> | Study of a literacy project aimed at women dairy farmers |
| Geert Driessen | 2007 | The feminization of primary education: Effects of teachers’ sex on pupil achievement, attitudes and behaviour*. IRE, 53*(2), 183–203. <https://doi.org/10.1007/s11159-007-9039-y> | Efforts to recruit mor men into teaching |
| Alan Rogers | 2007 | Women, literacy and citizenship: A critique*. IRE, 53*(2), 159–181 (2007). <https://doi.org/10.1007/s11159-006-9031-y> | Deepening literacy and citizenship education for women |
| Jennifer Shindler& Brahm Fleisch | 2007 | Schooling for all in South Africa: Closing the gap*. IRE, 53*(2), 135–157. <https://doi.org/10.1007/s11159-007-9038-z> | Gender-disaggregated statistics by province |
| Samir Ranjan Nath | 2007 | Self-reporting and test discrepancy: Evidence from a national literacy survey in Bangladesh*. IRE, 53*(2), 119–133. <https://doi.org/10.1007/s11159-007-9037-0> | Gender-disaggregated data on self report and literacy attainment |
| Margrit Stamm | 2007 | Begabung, Leistung und Geschlecht: Neue Dimensionen im Lichte eines alten erziehungswissenschaftlichen Diskurses [Giftedness, achievement and gender: New dimensions in light of an old educational discourse]*. IRE, 53*(4), 417–437. <https://doi.org/10.1007/s11159-007-9050-3> | Giftedness, gender and achievement |
| Jackie Kirk & Rebecca Winthrop | 2007 | Promoting quality education in refugee contexts: Supporting teacher development in northern Ethiopia*. IRE,* 53(5–6), 715–723. <https://doi.org/10.1007/s11159-007-9061-0> | Some reports of the experience of women teachers |
| Agneta Lind | 2008 | Literacy programmes for adults: What can we expect? *IRE,* 54(5–6), 755–761. <https://doi.org/10.1007/s11159-008-9103-2> | Some reflection on gender issues |
| Christine Fourner, Pierre Béret, Pierre Doray & Paul Bélanger | 2009 | Entre reproduction et mobilisation: les rapports de genre en formation continue en France et au Canada [Reproduction or mobilisation? Gender proportions in continuing education in France and Canada]*. IRE, 55*(1), 75–103. <https://doi.org/10.1007/s11159-008-9117-9> | Greater participation of women in CPD classes |
| Evelin G. Lindner | 2009 | Why there can be no conflict resolution as long as people are being humiliated*. IRE, 55*(2–3), 157–181. <https://doi.org/10.1007/s11159-008-9125-9> | Discussed gender as part of human rights and thinking about equality |
| Birgit Brock-Utne | 2009 | A gender perspective on peace education and the work for peace*. IRE, 55*(2–3), 205–220. <https://doi.org/10.1007/s11159-008-9122-z> | War as a masculinist activity |
| Marie Huet-Gueye & Myriam de Léonardis | 2009 | L’école publique au Sénégal : Approche psychosociale des pratiques parentales de (non) scolarisation et des expériences éducatives des enfants [Public schools in Senegal: Analysing the reasons for (non) enrolment ]*. IRE, 55*(4), 367–391. <https://doi.org/10.1007/s11159-009-9134-3> | Study of parent–child relationships in Senegal and attitudes to schooling. Data deals with gender although this is not picked up much in discussion |
| Samuel M. Davidson | 2009 | Mouths wide shut: Gender-quiet teenage males on gender-bending, gender-passing and masculinities*. IRE, 55*(5–6), 615–631. <https://doi.org/10.1007/s11159-009-9139-y> | Undoing gender – masculinities |
| Greg Knotts | 2009 | Undoing gender through legislation and schooling: The case of AB 537 and AB 394 in California, USA*. IRE, 55*(5–6), 597–614. <https://doi.org/10.1007/s11159-009-9138-z> | Undoing gender – administration |
| Sondra Cuban | 2009 | “Talking was a great experience”: Destabilising gendered communication in the workplace*. IRE, 55*(5–6), 579–596. <https://doi.org/10.1007/s11159-009-9144-1> | Undoing gender. England car workers |
| Barbara Ann Cole | 2009 | Gender, narratives and intersectionality: Can Personal experience approaches to research contribute to “undoing gender”? *IRE, 55*(5–6), 561–578. <https://doi.org/10.1007/s11159-009-9140-5> | Narrative method for undoing gender |
| Golnar Mehran | 2009 | “Doing and undoing gender”: Female higher education in the Islamic republic of Iran*. IRE, 55*(5–6), 541–559. <https://doi.org/10.1007/s11159-009-9145-0> | Addressing male disempowerment as well as exclusions of women |
| Sherrie Carinci & Pia Lindquist Wong | 2009 | Carinci, S., Wong, P.L. Does gender matter? An exploratory study of perspectives across genders, age and education*. IRE*, 55(5–6), 523–540. <https://doi.org/10.1007/s11159-009-9141-4> | Survey of attitudes to gender. USA? |
| Erin Murphy-Graham | 2009 | Constructing a New Vision: Undoing Gender through Secondary Education in Honduras*. IRE, 55*(5–6), 503–521. <https://doi.org/10.1007/s11159-009-9143-2> | Test scores and gender consciousness |
| Monisha Bajaj | 2009 | Un/doing gender? A case study of school policy and practice in Zambia*. IRE, 55*(5–6), 483–502. <https://doi.org/10.1007/s11159-009-9142-3> | A school’s policy and practice undoing gender |
| Nelly P. Stromquist & Gustavo E. Fischman | 2009 | Stromquist, N.P., Fischman, G.E. Introduction – From Denouncing Gender Inequities to Undoing Gender in Education: Practices and Programmes Toward Change in the Social Relations of Gender*. IRE, 55*(5–6), 463–482. <https://doi.org/10.1007/s11159-009-9146-z> | Definitions and approaches to gender |
| Yuko Nonoyama- Tarumi, Edilberto Loaiza & Patrice L. Engle | 2010 | Late entry into primary school in developing societies: Findings from cross-national household surveys*. IRE, 56*(1), 103–125. <https://doi.org/10.1007/s11159-010-9151-2> | Gender & mothers’ education as variables in age of entry into schooling |
| Ishmael I. Munene & Sara J. Ruto | 2010 | The right to education for children in domestic labour: Empirical evidence from Kenya*. IRE, 56*(1), 127–147. <https://doi.org/10.1007/s11159-010-9152-1> | Girls working in domestic labour |
| Mariangeles de la Caba Collado & Isabel Bartau Rojas | 2010 | Educational interventions targeted at minors in situations of grave social vulnerability and their families*. IRE, 56*(4), 377–398. <https://doi.org/10.1007/s11159-010-9171-y> | Intervention with a family looking at mothers and fathers |
| Steve Alsop, Patricia Ames, Graciela Cordero Arroyo & Don Dippo | 2010 | Programa de fortalecimiento de capacidades: Reflections on a case study of community-based teacher education set in rural northern Peru*. IRE, 56*(5–6), 633–649. <https://doi.org/10.1007/s11159-010-9178-4> | Reports on a project with a woman teacher countering machismo |
| Shirley Walters & Linda Cooper | 2011 | Learning/work: Turning work and lifelong learning inside out*. IRE, 57*(1–2), 27–38. <https://doi.org/10.1007/s11159-011-9189-9> | Gender as part of intersectionality in understanding power associated with skills and work |
| Carolyn Medel-Añonuevo & Anna Bernhardt | 2011 | Sustaining advocacy and action on women’s participation and gender equality in adult education*. IRE, 57*(1–2), 57–68. <https://doi.org/10.1007/s11159-011-9212-1> | Women’s movements and gender equality in adult ed |
| Goli M. Rezai-Rashti & Valentine M. Moghadam | 2011 | Women and higher education in Iran: What are the implications for employment and the “marriage market”? *IRE, 57*(3–4), 419–441 (2011). <https://doi.org/10.1007/s11159-011-9217-9> | Gender relationships |
| Nagwa Megahed & Stephen Lack | 2011 | Colonial legacy, women’s rights and gender-educational inequality in the Arab World with particular reference to Egypt and Tunisia*. IRE, 57*(3–4), 397–418. <https://doi.org/10.1007/s11159-011-9215-y> | Gender relations |
| Grace Feuerverger | 2011 | Re-bordering spaces of trauma: auto-ethnographic reflections on the immigrant and refugee experience in an inner-city high school in Toronto*. IRE,* 57(3–4), 357–375. <https://doi.org/10.1007/s11159-011-9207-y> | Intersectional approach |
| Amita Chudgar, Karyn Miller & Brij Kothari | 2012 | Relationship between household literacy and educational engagement: Analysis of data from Rajkot district, India*. IRE, 58*(1), 73–89. <https://doi.org/10.1007/s11159-012-9261-0> | Gender, education levels and household attitudes |
| Asako Yoshino | 2012 | The relationship between self-concept and achievement in TIMSS 2007: A comparison between American and Japanese students*. IRE, 58*(2), 199–219. <https://doi.org/10.1007/s11159-012-9283-7> | Family relationships and parental level of education |
| Casandra M. Guariono & Jeffery C. Tanner | 2012 | Adequacy, accountability, autonomy and equity in a Middle Eastern school reform: The case of Qatar*. IRE, 58*(2), 221–245. <https://doi.org/10.1007/s11159-012-9286-4> | Gender and planning |
| Cristine A. Smith, Rebecca Paulson Stone & Sarah Kahando | 2013 | A model of women’s educational factors related to delaying girls’ marriage*. IRE, 58*(4), 533–555. <https://doi.org/10.1007/s11159-012-9309-1> | Gender relations beyond school |
| Hadrat Yusif, Ishak Yussof & Zulkifly Osman | 2013 | Public university entry in Ghana: Is it equitable? *IRE, 59*(1), 7–27. <https://doi.org/10.1007/s11159-013-9331-y> | Gender as a variable in university admissions |
| Takako Yuki, Keiko Mizuno, Keiichi Ogawa & Sakai Mihoko | 2013 | Promoting gender parity in basic education: Lessons from a technical cooperation project in Yemen*. IRE, 59*(1), 47–66. <https://doi.org/10.1007/s11159-013-9341-9> | Girls’ access to school |
| Geert Driessen & Annemarie van Langen | 2013 | Gender differences in primary and secondary education: Are girls really outperforming boys? *IRE, 59*(1), 67–86. <https://doi.org/10.1007/s11159-013-9352-6> | Gender and learning outcomes |
| Valérie Delaunay, Bénédicte Gastineau & Frédérique Andriamaro | 2013 | Statut familial et inégalités face à la scolarisation à Madagascar [The impact of family status in Madagascar on inequalities in schooling]*. IRE, 59*(6), 669–692 (2013). <https://doi.org/10.1007/s11159-013-9388-7> | Family gender relationships and dropout |
| Makram Zghibi, Hajer Sahli, Mohamed Jabri, Samira Ouelhezi, Noomen Guelmemi & Nathalie Wallian | 2013 | Modalités de prise de décision chez les filles et les garçons en la présence ou en l’absence de l’enseignant [How girls and boys take decisions in the presence and absence of a teacher]*. IRE, 59*(6), 751–769. <https://doi.org/10.1007/s11159-013-9397-6> | Decisions about participation in sport between girls and boys |
| Sedat Gumus | 2014 | The effects of community factors on school participation in Turkey: A multilevel analysis*. IRE*, *60*(1), 79–98. <https://doi.org/10.1007/s11159-014-9411-7> | Analysis of community factors in children out of school |
| Birger Fredriksen & Camilla Helgø Fossberg | 2014 | The case for investing in secondary education in sub-Saharan Africa (SSA): Challenges and opportunities*. IRE, 60*(2), 235–259. <https://doi.org/10.1007/s11159-014-9407-3> | Gendered patterns of distribution; who is in lower secondary school |
| Nelly P. Stromquist | 2014 | Freire, literacy and emancipatory gender learning*. IRE*, 60(4), 545–558. <https://doi.org/10.1007/s11159-014-9424-2> | Gender and emancipation |
| Jamil Salmi & Roberta Malee Bassett | 2014 | The equity imperative in tertiary education: Promoting fairness and efficiency*. IRE, 60*(3), 361–377. <https://doi.org/10.1007/s11159-013-9391-z> | Gender distribution higher ed |
| Caitlin S. Haugen, Steven J. Klees, Nelly P. Stromquist, Jing Lin, Truphena Choti & Carol Corneilse | 2014 | Increasing the number of female primary school teachers in African countries: Effects, barriers and policies*. IRE, 60*(6), 753–776. <https://doi.org/10.1007/s11159-014-9450-0> | Gender and teachers’ work |
| Barbara Crossouard & Máiréad Dunne | 2015 | Politics, gender and youth citizenship in Senegal: Youth policing of dissent and diversity*. IRE, 61*(1), 43–60. <https://doi.org/10.1007/s11159-015-9466-0> | Vulnerability around reproductive rights in political activism |
| Bengü Börkan, Özlem Ünlühisarcıklı, H. Ayşe Caner & Z. Hande Sart | 2015 | The catch-up education programme in Turkey: Opportunities and challenges*. IRE, 61*(1), 21–41. <https://doi.org/10.1007/s11159-015-9464-2> | Gender distribution dropout |
| Malini Ghose & Disha Mullick | 2015 | A tangled weave: Tracing outcomes of education in rural women’s lives in North India*. IRE, 61*(3), 343–364. <https://doi.org/10.1007/s11159-014-9449-6> | Literacy & learning outcomes |
| Bénédicte Gastineau, Norbert Kpadonou, Valérie Delaunay, Eve Senan Assogba & Josette Gnélé | 2015 | Inégalités scolaires à Cotonou (Bénin) : rôle croissant de l’encadrement familial à la recherche d’une éducation de qualité [Educational inequality in Cotonou (Benin): The increasing role of family supervision in obtaining quality education]*. IRE, 61*(4), 445–464 (2015). <https://doi.org/10.1007/s11159-015-9502-0> | Interviews with women about schooling |
| Taeko Takayanagi | 2016 | Rethinking women’s learning and empowerment in Kenya: Maasai village women take initiative*. IRE, 62*(6), 671–688. <https://doi.org/10.1007/s11159-016-9597-y> | Empowerment |
| Joséphine Wouango | 2017 | L’accès des filles à l’enseignement supérieur au Burkina Faso: choix parentaux, parcours d’étudiantes et défis [Girls’ access to higher education in Burkina Faso: parental choices, student trajectories, challenges]*. IRE, 63*(2), 213–233. <https://doi.org/10.1007/s11159-016-9610-5> | Girls’ access to HE |
| Johanna Ennser-Kananen & Nicole Pettitt | 2017 | “I want to speak like the other people”: Second language learning as a virtuous spiral for migrant women? *IRE, 63*(4), 583–604. <https://doi.org/10.1007/s11159-017-9653-2> | Adult women learners |
| Turuwark Zalalam Warkineh, Alan Rogers & Tolera Negassa Danki | 2018 | Profiling adult literacy facilitators in development contexts: An ethnographic study in Ethiopia*. IRE, 64*(1), 9–30. <https://doi.org/10.1007/s11159-017-9686-6> | Gender experiences of adult literacy facilitators |
| Suehye Kim | 2018 | Literacy skills gaps: A cross-level analysis on international and intergenerational variations*. IRE, 64*(1), 85–110. <https://doi.org/10.1007/s11159-018-9703-4> | Gender and literacy distribution |
| Birgit Brock-Utne | 2018 | Researching language and culture in Africa using an autoethnographic approach*. IRE, 64*(6), 713–735. <https://doi.org/10.1007/s11159-018-9746-6> | Methodology |
| Torill Aagot Halvorsen | 2018 | My face is more than me. A Nordic researcher in the South: An autoethnographic retrospective perspective*. IRE, 64*(6), 845–864. <https://doi.org/10.1007/s11159-018-9744-8> | Methodology; insider/outsider |
| Matthew A. Witenstein & Radhika Iyengar | 2021 | A bottom-up approach to improve women’s access to technical and vocational education and training in India: Examining a non-formal education upskilling programme*. IRE, 67*(4), 475–484. <https://doi.org/10.1007/s11159-021-09890-1> | Adult women learning |
| Marcia Kim & Yan Guo | 2021 | Resisting the soft skills discourse: Perspectives and experiences of internationally educated nurses in Canada*. IRE, 67*(6), 751–770. <https://doi.org/10.1007/s11159-021-09934-6> | Comments on experience of work and training but no comments on gender. All women in study |
| Suzanne Smythe | 2022 | The Faure report, Sylvia Wynter and the undoing of the Man of lifelong learning*. IRE, 68*(5), 773–789. <https://doi.org/10.1007/s11159-022-09980-8> | Conceptual |
| Alla Rastrygina & Nadiya Ivanenko | 2023 | A pedagogy of freedom as a viable basis for implementing gender equality in Ukraine’s educational institutions*. IRE, 69*(1–2), 143–174. <https://doi.org/10.1007/s11159-023-09995-9> | Gender equality in education |
| Jinhee Choi & Esther Prins | 2023 | Choi, J., Prins, E. North Korean women entrepreneurs learning from failure*. IRE, 69*(1–2), 207–226. <https://doi.org/10.1007/s11159-023-09994-w> | Adult learners |
| Ayhan Görmüş & Meryem Baytur | 2023 | Distance education and work–family conflict during COVID-19: Evidence from Turkey for a gender-moderated model*. IRE, 69*(5), 625–649. <https://doi.org/10.1007/s11159-023-10030-0> | Family gender relations |
| Jacob Chomba Nshimbi & Robert Serpell | 2023 | Growth and application of literacy skills by rural Zambian mothers with assistance from their children*. IRE, 69*(6), 795–822. <https://doi.org/10.1007/s11159-023-10028-8> | Mothers’ literacy |
| Bianca Rochelle Parry | 2024 | “We’ve got a sisterhood …”: Understanding personal and peer empowerment capabilities in the narratives of South African women furthering their education while incarcerated*. IRE, 70*(3), 417–431. <https://doi.org/10.1007/s11159-023-10051-9> | Adult women learners |
| Elaine Unterhalter | 2024 | Soft power in complicated and complex education systems: Gender, education and global governance in organisational responses to SDG 4*. IRE, 70*(4), 547–573. <https://doi.org/10.1007/s11159-024-10098-2> | Study of global policy |
